# Community-onset symptomatic urinary tract infections (SUTI) caused by extended-spectrum beta-lactamase (ESBL)-producing Enterobacterales: independent predictors and comparative effectiveness of oral agents

**DOI:** 10.1017/ash.2025.10267

**Published:** 2026-01-08

**Authors:** Shani Zilberman-Itskovich, Majdi Masarwi, Eyal Levy, Moti Iflah, Inbar Levi Steinweg, Nikita Yapryntsev, Shani Mednyk, Roni Gur-Lavy, Samir Alfahel, Keren Amity, Avi Itzhaki, Dror Marchaim

**Affiliations:** 1 Shamir (Assaf Harofeh) Medical Center, Zerifin, Israel; 2 Faculty of Medicine, Tel Aviv University, Tel-Aviv, Israel

## Abstract

**Background:**

The incidence of community-onset (CO) symptomatic urinary tract infection (SUTI) caused by extended-spectrum beta-lactamase (ESBL)-producing Enterobacterales is increasing worldwide. Our study aims were to explore the independent predictors for CO-ESBL SUTI and to compare the effectiveness of several oral therapeutics, which are used for this indication in community health settings.

**Methods:**

Retrospective matched case-case-control and case-case studies, among insurers of Maccabi health maintenance organization, Shfella district, Israel (10–11/2019). Patients with CO-ESBL (*Escherichia coli, Klebsiella pneumoniae, Proteus mirabilis*) SUTI were matched to patients with CO-non-ESBL SUTI and to uninfected controls (1:1:1). Matched analyses (logistic regressions) were used to model predictors for CO-ESBL SUTI. A composite parameter for worse SUTI outcomes was compared among patients who were managed with a single, supposedly effective (ie, in vitro), oral agent.

**Results:**

The study consisted of 1,455 patients (ie, three matched groups of 485 patients). The independent predictors for CO-ESBL SUTI were certain recent exposures: (1) hospitalization (3 months), (2) past carriage of multidrug-resistant organisms (2 years), (3) exposure to any antimicrobial (3 months), and (4) prior SUTI (6 months). Among 331 patients with CO-ESBL SUTI, resistance rates were lowest for fosfomycin (4.9%), while outcomes were worst for patients managed with oral amoxicillin-clavulanate.

**Conclusions:**

CO-ESBL SUTI independent predictors in this community region were recent hospitalization, known MDRO carriage, exposure to antimicrobials and prior SUTI. Amoxicillin-clavulanate should be avoided, even for ESBL susceptible isolates.

## Introduction

Symptomatic urinary tract infection (ie, SUTI^1^) is a common bacterial infection, occurring both in community health settings and in acute-care hospitals.^2^ The severity of SUTI range from mild, simple cystitis, to a severe life-threatening systemic infection.^2,3^ More than 90% of SUTI offending pathogens, belong to the Enterobacterales family (eg, *Escherichia coli*).^2,3^ Extended spectrum beta-lactamase (ESBL) production among community-onset (CO) Enterobacterales isolates- particularly *E. coli*, *Klebsiella pneumoniae*, and *Proteus mirabilis* -had become endemic worldwide.^4^ In 2024 the World Health Organization (WHO) classified these ESBL-producing Enterobacterales as “priority pathogens,” underscoring their global burden and threat on public health.^5^ Despite this statement, controlled evidence from the community health settings, regarding the epidemiology of ESBL infections, remains limited.^6–8^ This is especially evident in mild SUTI, a common infectious syndrome that is often managed exclusively in the community.

In controlled trials, which were executed primarily at acute-care hospitals, not in community settings, invasive ESBL infections were significantly associated with worse patients’ outcomes (in comparison to patients with Enterobacterales susceptible infections, and in comparison to uninfected controls), primarily resulting from delays in initiation of appropriate antimicrobial therapy (DAAT).^9,10^ DAAT is a strong modifiable independent predictor of mortality in severe sepsis,^11^ and specifically in ESBL infections.^12^ Although its impact on outcomes in milder infections may be less pronounced,^11^ identifying independent predictors of CO-ESBL SUTI—even in mild cases—could improve empiric prescribing practices and patient outcomes.

Matched case-case-control design is the gold standard methodology to determine independent predictors for acquisition of a multidrug-resistant organism (MDRO) in general,^13^ and specifically of ESBL infections^14^ in hospitals. In the community, it is less established which patients should represent the source population from which the ESBL SUTI cases had “epidemiologically evolved.” Currently, there is lack in both case-case-control studies (ie, using a group of matched uninfected controls to reflect the source population) and lack of case-case studies (ie, using a group of matched patients with non-ESBL SUTI to reflect the source population) to explore the independent predictors of CO-ESBL SUTI managed in community settings.^15,16^

The Infectious Diseases Society of America (IDSA) recently revised its treatment guidelines for ESBL-producing Enterobacterales infections.^17^ Nitrofurantoin and trimethoprim-sulfamethoxazole (TMP-SMX) are recommended as preferred oral options for mild ESBL cystitis, provided the isolates are susceptible.^17^ The IDSA discourages the use of oral fluoroquinolones (eg, ciprofloxacin, levofloxacin) for this indication due to resistances and adverse events.^17^ Oral amoxicillin–clavulanate is also strongly discouraged;^17^ however, because it often appears on microbiology reports as a theoretically “valid” oral beta-lactam option, it may still be prescribed by non-specialists when isolates show phenotypic *in-vitro* susceptibility.^18^ Fosfomycin is another oral option mentioned in the IDSA guidelines, but due to the current US Food and Drug Administration (FDA) and Clinical and Laboratory Standards Institute (CLSI) registration restrictions and lack of breakpoints, fosfomycin is used in the United States. only for uncomplicated *E. coli* cystitis, in a single oral dose of three grams (regardless of ESBL production).^17^ In several countries outside the United States, including in Israel, oral fosfomycin is administered for mild ESBL cystitis in three repeated doses of three grams, for three consecutive days, although controlled data pertaining to the effectiveness of this regimen are scarce.^19^

The aims of our study were: (1) to identify the independent predictors of CO-ESBL SUTI, and (2) to compare the effectiveness of oral regimens used for this indication. This could improve empiric prescribing (aim 1) and therapeutic management (aim 2) of ESBL SUTI in community health settings.

## Methods

A retrospective observational matched case-case-control study^13^ and a matched case-case study were conducted among insurers of Maccabi healthcare health maintenance organization (HMO), from the Shfella district, Israel, for two consecutive months (10–11/2019). Maccabi health care is the second largest HMO in the country, with over 600,000 insurers listed to the Shfella district. All positive urine cultures were reviewed, defined according to the US Centers for Disease Control and Prevention (CDC) criteria, that is, as ≤2 pathogens and inoculum growth >10^5^ CFU/mL^1^. Unique adult patients (>18 years) were included, who had either *E. coli*, *K. pneumoniae*, or *P. mirabilis* CO SUTI, determined according to CDC definitions.^1^ Patients were included only if they had symptomatic UTI (eg, dysuria, high frequency, suprapubic pain or urgency, with or without fever and hematuria).^20,21^ Patients with asymptomatic bacteriuria were excluded. Complicated UTI (cUTI) was determined according to IDSA definition.^22^ ESBL was diagnosed using the automated VITEK-2 (*bioMérieux* Inc., France), in accordance with CLSI 2019 criteria and breakpoints.^23^ Patients with carbapenem-resistant and/or carbapenemase-producing Enterobacterales were excluded. The HMO ethic committee had approved the study prior its initiation.

The study comprised of three groups: (1) patients with CO-ESBL SUTI, (2) Patients with CO-non-ESBL SUTI, and (3) Uninfected controls without SUTI or any other active infection.^13^ If ESBL was isolated in a polymicrobial culture, the patient was considered an ESBL “case.” Matching patients with CO-ESBL SUTI to patients with CO-non-ESBL SUTI (from the same 2-month study period) was in accordance with three parameters (listed in order of importance^24^): (1) bacteria type, (2) age group (in decades), and (3) place of residency (home versus any long-term care facility [LTCF]). The last criterion reflects the ambulatory equivalent to the parameter “time at risk,” to avoid over-matching for certain potential important predictors (eg, female sex, acute illness indices), in accordance with the established case-case-control methodology.^24^ Matching patients with CO-ESBL SUTI to uninfected controls for the case-case-control study was in accordance (listed in order of importance^24^): (1) age group (in decades), (2) place of residency (home versus LTCF), and (3) if they visited the same family clinic, at the same day (for a non-infectious complaint), as their matched ESBL case.

Since it is difficult to determine in the ambulatory settings the “appropriate” source population from which patients with ESBL SUTI had “epidemiologically evolved,” we determined the independent predictors for CO-ESBL SUTI in two separate analyses. First, is the “classical” case-case-control design,^24^ that is, the eventual predictors would be those associated with ESBL SUTI in its model versus uninfected controls (model 1), but which are not associated as well, with CO-non-ESBL SUTI in its model versus uninfected controls (model 2). The second analyses would be a case-case study, that is, the eventual predictors would be those associated with ESBL SUTI in its direct comparison to patients with non-ESBL SUTI (model 3).

The comparative effectiveness analyses of the various oral agents (study aim 2), were determined according to the outcomes of CO-ESBL SUTI patients who were treated with a single oral agent (for up to 14 days), for which their initial ESBL isolate was susceptible (in vitro^23^). Since the study focused on patients with milder SUTI who were managed in the community (at least initially) with oral agents, patients who were immediately referred during the initial SUTI visit to the emergency room, or to home parenteral therapy (through a home IV team), were excluded (but were included in the ESBL predictors’ analyses, that is, study aim (1). However, referral to hospital or to home IV following the initial visit were captured as treatment failures. The oral regimens for ESBL SUTI that were compared (ie, embedded in the HMO electronic medical records system, to be used according to prescriber’s discretion): (1) nitrofurantoin (300 mg daily administered for 5–7 days), (2) TMP-SMX (for 5–7 days), (3) fluroquinolone (ciprofloxacin, levofloxacin, or ofloxacin for up to 7 days), (4) fosfomycin (3g daily, administered for 3 consecutive days), and (5) amoxicillin-clavulanate (875 mg amoxicillin/125 mg clavulanic acid twice daily for 7 days). Since SUTI is a mild condition with difficulties to determine the outcome based on retrospective charts review, we created a composite outcome to determine treatment failure. The composite outcome was met, if in the 14 days following the initial SUTI visit, patients had experienced any of the following: (1) clinical failure (documentation of continued SUTI symptoms or complains), (2) bacteriological failure (additional positive urine culture/s), (3) referral to emergency room or to home IV, (4) hospitalization, or (5) death (any cause).

Statistical analyses were executed with IBM SPSS 29.0 (2024). Univariable matched analyses, followed by multivariable matched analyses (logistic regression, stepwise backward selection), were executed to determine the independent predictors for CO-ESBL SUTI by comparing patients with CO-ESBL SUTI to uninfected controls (model 1), patients with CO-non-ESBL SUTI to uninfected controls (model 2),^13^ and patients with CO-ESBL SUTI to patients with CO-non-ESBL SUTI (model 3). Predictors that were independently associated with CO-ESBL SUTI in model 1 but not with CO-non-ESBL SUTI in model 2 (case-case-control design), and the independent predictors per model 3 (case-case design), were considered potential independent predictors for CO-ESBL SUTI^13^ (study aim 1). Variables with significant association (*P* < .05) per univariable analyses, were incorporated to the multivariable models. Models were tested for confounding and collinearity.^25^ SUTI acute illness indices, DAAT, and categorical and time-dependent outcomes, were compared only between the patients with CO-ESBL SUTI and the patients with CO-non-ESBL SUTI, using logistic and Cox regressions, respectively. χ2 or Fisher’s exact tests were used to compare the effectiveness of the various oral agents, versus categorical outcomes, including the composite outcome (study aim 2).

## Results

### Descriptive analyses

There were 13,597 positive urine cultures^1^ of Enterobacterales (*E. coli*, *K. pneumoniae*, and *P. mirabilis* only) during the 2-month study period. After applying all inclusion criteria as depicted above, 1,455 unique patients were included in the final cohort, divided to three perfectly matched groups of 485 patients (Table 1): patients with CO-ESBL SUTI, patients with CO-non-ESBL SUTI, and uninfected controls. None of the patients died (of any cause) in the 14 days following the index visit. The mean population’s age was 58 ± 19 years and 1,144 (79%) were females. At baseline, 96 patients (6.6%) were functionally dependent,^26^ 36 (2.5%) were chronic LTCF residents, and 22 patients (1.5%) had a urinary catheter. There were 446 (31%) patients with documentation of recent SUTI (in the previous 6 months), 557 (38%) had received antibiotics in the previous 3 months (for any indication), and 185 (12.7%) patients were known ESBL carriers from the previous year. The median Charlson’s weighted index comorbidity for the entire population was 1 (IQR = 0–2).

**Table 1.** Univariable comparisons between patients with CO-ESBL SUTI, CO-non-ESBL SUTI, and uninfected controls (485 patients in each group), maccabi health care, 10–11/2019

Parameter		ESBL SUTI, N (%)^a^	Non-ESBL SUTI, N (%)^a^	Uninfected controls, N (%)^a^	ESBL SUTI versus uninfected controls, OR* (CI-95%), *P* value	Non-ESBL SUTI versus uninfected controls, OR* (CI-95%), *P* value	ESBL versus susceptible, OR* (CI-95%), *P* value
**Demographics**							
Age, years, mean ± SD		58 ± 19	58 ± 20	57 ± 19	*P* = .41	*P* = .43	*P* = .96
Elderly (>65 years old)		208 (43)	220 (45)	199 (41)	1.1 (0.8–1.4), *P* = .56	1.2 (0.9–1.5), *P* = .17	0.9 (0.7–1.2), *P* = .44
Male sex		67 (14)	42 (9)	202 (42)	**0.23 (0.2–0.3), *P* < .001**	**0.13 (0.1–0.2), *P* < .001**	**1.7 (1.1–2.5), *P* = .011**
**Recent exposures to healthcare environments/settings/procedures**							
Recent hospitalization in the prior 3 months		68 (14)	24 (4.9)	17 (3.5)	**4.5 (2.6–7.8), *P* < .001**	1.4 (0.8–2.7), *P* = .26	**3.1 (1.9–5.1), *P* < .001**
Advanced nursing care administered at home		5 (1)	6 (1.2)	0	1.01 (1–1.02), *P* = .06	***P* = .03**	0.8 (0.3–2.7), *P* = .8
Permanent device^b^		39 (8)	29 (6)	17 (3.5)	**2.4 (1.3–4.3), *P* = .002**	1.8 (0.95–3.2), *P* = .07	1.4 (0.8–2.3), *P* = .21
Urinary catheter		19 (3.9)	3 (0.6)	0	***P* < .001**	*P* = .25	**6.6 (1.9–22.3), *P* = .001**
Recent invasive procedure^c^		86 (18)	56 (11.5)	32 (6.6)	**3.1 (2–4.68), *P* < .001**	**1.9 (1.2–2.9), *P* = .007**	**1.7 (1.2–2.4), *P* = .006**
Recent urologic procedure in the prior 3 months		21 (4.3)	51 (10.5)	1 (0.2)	**22 (2.9–164), *P* < .001**	**57 (7.8–413), *P* < .001**	**0.4 (0.2–0.4), *P* < .001**
Recent pregnancy or delivery in the prior 3 months		7 (1.6)	2 (0.4)	0	***P* = .03**	*P* = .52	3.7 (.8–18), *P* = .1
**Recent exposures to antimicrobials in the prior 3 months**							
Any antibiotic		294 (61)	199 (41)	64 (13.2)	**10.1 (7.4–13.9), *P* < .001**	**4.6 (3.3–6.3), *P* < .001**	**2.1 (1.7–2.9), *P* < .001**
Penicillin/s		49 (10)	28 (6)	11 (2.3)	**4.8 (2.5–9.4), *P* < .001**	**2.6 (1.3–5.4), *P* = .005**	**1.8 (1.1–3), *P* = .013**
BLBLI		50 (10)	22 (4.5)	9 (1.9)	**6.1 (3–12.5), *P* < .001**	**2.5 (1.2–5.5), *P* = .018**	**2.4 (1.4–4.1), *P* = .001**
Cephalosporin		120 (25)	54 (11)	4 (0.8)	**40 (15–108), *P* < .001**	**15.1 (5.4–42), *P* < .001**	**2.6 (1.9–3.7), *P* < .001**
Flouroquinolone		124 (26)	55 (11)	5 (1)	**33 (13.3–82), *P* < .001**	**12.3 (4.9–31), *P* < .001**	**2.7 (1.9–3.8), *P* < .001**
Aminoglycoside		12 (2.5)	0	0	***P* < .001**		***P* < .001**
TMP-SMX		33 (7)	9 (2)	1 (0.2)	**35 (4.8–259), *P* < .001**	**9.2 (1.2–73), *P* = .021**	**3.9 (1.8–8.2), *P* < .001**
Nitrofurantoin		49 (10)	34 (7)	2 (0.4)	**27 (6.6–112), *P* < .001**	**18.2 (4.4–76), *P* < .001**	1.5 (0.94–2.4), *P* = .085
Fosfomycin		63 (13)	50 (10)	6 (1.2)	**12 (5.1–28), *P* < .001**	**9.2 (3.9–22), *P* < .001**	1.3 (.9–1.4), *P* = .2
Macrolide		45 (9.3)	8 (1.6)	10 (2.1)	**4.9 (2.4–9.8), *P* < .001**	0.8 (0.31–2.04), *P* = .63	**6.1 (2.8–13.1), *P* < .001**
Metronidazole		10 (2.1)	2 (0.4)	0	***P* = .002**	*P* = .5	**5.1 (1.1–23), *P* = .04**
**Background medical states / conditions**							
Dependent functional status		48 (9.9)	32 (6.6)	61 (3.3)	**3.2 (1.8–5.8), *P* < .001**	**2.1 (1.1–3.8), *P* = .018**	1.6 (0.98–2.45), *P* = .06
Consciousness/cognitive impairment in background		19 (3.9)	17 (3.5)	2 (0.4)	**9.9 (2.9–43), *P* < .001**	**8.8 (2–38), *P* = .001**	1.1 (0.6–2.2), *P* = .7
Recent SUTI in prior 6 months		223 (46)	182 (38)	41 (8.5)	**9.1 (6.3–13.2), *P* < .001**	**6.5 (4.5–9.4), *P* < .001**	**1.4 (1.1–1.8), *P* = .008**
Days from last SUTI, mean ± SD		58 ± 46	67 ± 49	95 ± 51	***P* < .001**	***P* = .001**	*P* = .06
Past MDRO in the prior 12 months^d^		112 (23)	50 (10.3)	3 (0.6)	**48 (15–153), *P* < .001**	**19 (5.7–60), *P* < .001**	**2.6 (1.8–3.8), *P* < .001**
Past ESBL in the prior 12 months		115 (24)	60 (12)	10 (2.1)	**15 (7.6–29), *P* < .001**	**6.7 (3.4–13.3), *P* < .001**	**2.2 (1.6–3.1), *P* < .001**
Congestive heart failure		8 (1.6)	20 (4.1)	9 (1.9)	0.9 (0.3–2.3), *P* = .8	**2.3 (1.03–5.1), *P* = .04**	**0.4 (0.2–0.9), *P* = .02**
Diabetes mellitus		101 (21)	85 (18)	95 (20)	1.1 (0.8–1.5), *P* = .63	0.9 (0.6–1.2), *P* = .4	1.3 (0.9–1.7), *P* = .17
Chronic renal disease^e^		18 (3.7)	29 (6)	17 (3.5)	1.1 (0.5–2.1), *P* = .9	1.8 (0.95–3.2), *P* = .07	0.6 (0.3–1.1), *P* = .1
Chronic lung disease^f^		62 (13)	34 (7)	48 (10)	1.3 (0.9–2), *P* = .16	0.7 (0.4–1.1), *P* = .11	**1.9 (1.3–3), *P* = .003**
Connective tissue disease^g^		31 (6.4)	15 (3.1)	18 (3.7)	1.8 (0.98–3.1), *P* = .06	0.8 (0.4–1.7), *P* = .6	**2.1 (1.1–4), *P* = .016**
Dementia		15 (3.1)	14 (2.9)	1 (0.2)	**15.5 (2–117), *P* < .001**	**14 (1.9–110), *P* = .001**	1.1 (0.5–2.3), *P* = .85
Malignant tumor (past or active)		59 (12)	74 (15)	35 (7)	**1.8 (1.2–2.8),** ***P* = .01**	**2.3 (1.5–3.5), *P* < .001**	0.8 (0.5–1.1), *P* = .16
Chalrlson’s scores,^23^ median (IQR)	Weighted index comorbidity	1 (0–2)	1 (0–2)	0 (0–4)	***P* < .001**	***P* < .001**	*P* = .7
	Combined condition score	2 (1–4)	2 (1–4)	4 (1–7)	***P* < .001**	***P* < .001**	*P* = .9
	10-year survival probability, percent	87 (45–97)	87 (44–97)	57 (1–97)	***P* < .001**	***P* < .001**	*P* = .9
Glucocorticoids recent usage^h^		20 (4.1)	20 (4.1)	3 (0.6)	**6.9 (2–23),** ***P* < .001**	**6.9 (2–23),** ***P* < .001**	1 (0.5–1.9), *P* > .99
Immunosuppression / immunomodulation in the prior 3 months^i^		27 (5.6)	27 (5.6)	11 (2.3)	**2.5 (1.2–5.2), *P* = .008**	**2.5 (1.2–5.2), *P* = .008**	1 (0.6–1.7), *P* > .99

Notes. CO, community-onset; ESBL, extended-spectrum beta-lactamase-producing Enterobacterales; SUTI, symptomatic urinary tract infection; OR, odds ratio; CI, confidence intervals; SD, standard deviation; IQR, Interquartile range; MDRO, multidrug-resistant organism; BLBLI, beta-lactam beta-lactamase inhibitors combinations; TMP-SMX, trimethoprim-sulfamethoxazole; IDSA, Infectious Diseases Society of America; Dx, diagnosis; CDI, *Clostridioides difficile* infection.

a Valid percent: count divided by the total number of valid observations (after excluding the missing information from the denominator).

b Permanent devices: Patient has chronic/permanent device, that were in place at the date of SUTI initiation, eg., urinary catheter, tracheotomy, central line of any type, insulin pump, intra-uterine devices, orthopedic external fixators, implanted defibrillator, pacemaker, drains of any sort. Prosthetic heart valves, ileal conduit, joint prostheses, internal stents (eg, bile, coronary), were not captured as permanent devices.

c Past invasive procedure: Patient has had any type of invasive procedure in the prior six months, for example, any type of surgery (from minor to major), endoscopy, permanent central line insertion, lumbar puncture (LP), feeding tube insertion, abscess drainage, bone marrow biopsy, emergent dialysis.

d Past MDRO include any of the following: methicillin-resistant *Staphylococcus aureus* (MRSA), carbapenem-resistant or carbapenemase-producing Enterobacterales (CRE or CPE), vancomycin-resistant enterococci (VRE), ESBL-producing Enterobacterales, *Acinetobacter baumannii*, or *Pseudomonas aureginosa*.

e Chronic renal disease: serum creatinine≥1.7mg%.^28^

f Chronic lung diseases include chronic obstructive pulmonary disease (COPD), asthma, restrictive lung disease, pulmonary hypertension, bronchiectasis.

g Connective tissue disease include rheumatoid arthritis, systemic lupus erythematosus (SLE), scleroderma, vasculitis, psoriasis, gout, sarcoidosis.

h Glucocorticoids exposures were captured if administered for more than 48 hours in the past month.

i Immunosuppression / immunomodulation in the prior 3 months include any of the following: (1) neutropenia (<500 neutrophils) present at day of culture, (2) Glucocorticoid / steroid use for >48 hours in the past month, (3) Patient had received chemotherapy in the past 3 months, (4) Patient had received radiotherapy in the past 3 months, (5) Patient has HIV, (6) Patient has had a bone marrow or solid organ transplantation, and (7) Immunomodulators therapy in the past 3 months (eg, infliximab, adalimumab, certolizumab pegol, golimumab, etanercept).

Of the 970 SUTI patients depicted in Table 2 (ie, 485 patients with CO-ESBL SUTI and 485 patients with CO-non-ESBL SUTI), 736 patients (76%) had *E. coli*, 208 (21%) had *K. pneumoniae*, and 26 (3%) had *P. mirabilis*. There were 170 (17.5%) patients with cUTI.^17^ Overall, 841 (87%) patients had received appropriate antimicrobials, which was initiated in a median of one day (IQR = 0–4) from the index visit. There were 236 patients with documented clinical failure (ie, 24% of the entire SUTI cohort and 63% of the patients who had any additional documented visit to ambulatory clinic in the following 14 days) and 69 patients with documented bacteriological failure (ie, 7% of the entire SUTI cohort and 70% of the patients from which additional urine culture were obtained in the following 14 days). During the course of SUTI (up to day 14), 23 (2.4%) patients were referred to emergency rooms and 20 (2%) were hospitalized. Overall, 271 patients (28%) had met the composite worse outcome definition. As depicted in Table 2, Patients with ESBL SUTI suffered significantly from worse outcomes, that is, with higher rates of clinical failures, ER referrals, hospitalizations, and of the composite outcome. Of note, patients with non-ESBL SUTI had higher rates of bacteriological failures, though this was an insignificant statistical association.

**Table 2.** Antimicrobial management, acute illness indices, and outcomes, of patients with community-onset (CO) symptomatic urinary tract infections (SUTI), Maccabi health care, 10–11/2019 (n = 970)

Parameter	ESBL SUTI (n = 485), N (%)^a^	Non-ESBL SUTI (n = 485), N (%)^a^	ESBL versus susceptible, OR (CI-95%), *P* value
**Appropriate antimicrobial therapy**			
Days to appropriate therapy, median (IQR)	2 (0–4)	1 (0–2)	***P* < .001**
Appropriate therapy administered during SUTI (up to 14 days)	383 (80)	457 (94)	**0.3 (0.2–0.4), *P* < .001**
**Acute illness indices**			
Acute kidney injury^b^	5 (1)	4 (0.8)	1.25 (0.33–4.7), *P* > .99
Complicated UTI per IDSA definition^a^	80 (17)	90 (19)	0.9 (0.6–1.2), *P* = .4
Severe sepsis^27^	40 (8.2)	0	***P* < .001**
**Outcomes**			
Died in 14 days following SUTI Dx	0	0	
Died in 3 months following SUTI Dx	5 (1)	1 (0.2)	5 (0.6–13.3), *P* = .22
Bacteriological failure^c^	37 (64)	32 (46)	0.5 (0.2–1.2), *P* = .13
Clinical failure^d^	129 (69)	107 (58)	**1.6 (1.1–2.5), *P* = .03**
Emergency room referral in 14 days following SUTI Dx	17 (3.5)	6 (1.2)	**2.9 (1.1–7.4), *P* = .02**
Hospitalized in acute-care hospital in the 14 days following SUTI Dx	16 (3.3)	4 (0.8)	**4.1 (1.4–12.4), *P* = .011**
Composite end point^e^	150 (31)	121 (25)	**1.4 (1.1–1.8), *P* = .04**

Notes. ESBL, extended-spectrum beta-lactamase-producing Enterobacterales; SUTI, symptomatic urinary tract infection; OR, odds ratio; CI, confidence intervals; SD, standard deviation; IQR, Interquartile range; Dx, diagnosis.

a Valid percent: count divided by the total number of valid observations (after excluding the missing information from the denominator).

b Acute kidney injury was defined as a rise in serum creatinine by ≥1.5-fold.

c Bacteriological failure: continued positive urine culture within 14 days following culture date, only among patients that a culture was repeated.

d Clinical failure: continued SUTI’s symptoms or complains documented within 14 days following culture date.

e Composite end point was determined based of the presence of any one of the following parameters: bacteriological failure^b^, clinical failure^c^, referral to the emergency room, or had to be hospitalized.

### Predictors for CO-ESBL SUTI

The univariable comparisons between the three groups of patients are depicted in Table 1. Many of the epidemiological parameters were significantly associated with both CO-ESBL SUTI and CO-non-ESBL SUTI in comparisons versus uninfected controls: eg, female sex, certain background conditions (eg, recent prior SUTI, known carrier from the past year of ESBL or other MDRO, functionally dependent,^26^ cognitively impaired, malignant tumor, elevated Chalson’s scores,^23^ immunosuppressive conditions/states), and various recent healthcare exposures (eg, invasive procedure, urologic procedure, antibiotics in general and to certain classes in particular). However, there were certain epidemiological parameters, which were significantly associated only with CO-ESBL SUTI, but not with SUTI in general in the bivariable comparisons: eg recent hospitalization, permanent invasive device, the presence of a urinary catheter at the initial SUTI visit, and recent pregnancy or delivery. The epidemiological features that were associated with CO-non-ESBL SUTI (compared to uninfected controls), but not with CO-ESBL SUTI (compared to uninfected controls), were advanced nursing care administered at home, and congestive heart failure as a background condition (Table 1).

The bivariable analyses of the acute illness indices and of the outcomes, compared only the group of patients with CO-ESBL SUTI to the group of patients with CO-non-ESBL SUTI (Table 2). Patients with CO-ESBL SUTI had suffered significantly more often from severe sepsis indices,^27^ from DAAT, referrals to emergency rooms, hospitalizations during the course of SUTI, and from an overall increase rate of the composite outcome.

Table 3 summarize the final multivariable risk factors models for CO-ESBL SUTI versus uninfected controls (model 1), for CO-non-ESBL SUTI versus uninfected controls (model 2), and for CO-ESBL SUTI versus CO-non-ESBL SUTI (model 3). As highlighted in the table, the only parameter that was independently and significantly associated with model 1, but not with model 2 (ie, the case-case-control study), was recent (3 months) hospitalization in an acute-care facility. The independent predictors for CO-ESBL SUTI as per model 3 were again recent (3 months) hospitalization, in addition to carriage of MDRO from the previous two years, recent (3 months) exposure to (any) antimicrobial agent, and documented SUTI events from the past year (ie, the case-case study).

**Table 3.** Multivariable models of predictors for community-onset (CO) symptomatic urinary tract infections (SUTI), Maccabi health care, 10–11/2019

Variable	CO-ESBL SUTI vs. uninfected controls (model 1)		CO-non-ESBL SUTI vs uninfected controls (model 2)		CO-ESBL SUTI vs CO-non-ESBL SUTI (model 3)	
	Odds ratio (CI-95%)	*P*-value	Odds ratio (CI-95%)	Odds ratio (CI-95%)		
Female gender			5.6 (3.5–8.9)	<.001		
**Recent hospitalization (past 3 months)**	**4.8 (1.7–13.3)**	**.003**			**2.1 (1.2–3.8)**	**.01**
Known MDRO carrier (past 2 years)	21 (4.6–96)	<.001	10.8 (2.7–42)	<.001	2.9 (1.8–4.6)	<.001
Recent UTI (past 6 months)	2.7 (1.5–4.7)	<.001	3.5 (2.1–5.9)	<.001		
Recent urologic procedure (past 6 months)			44 (5.2–363)	<.001		
Recent exposure to antibiotic therapy (past 3 months)	4.7 (2.9–7.4)	<.001	2.2 (1.4–3.6)	.001	2.3 (1.8–3.1)	<.001
Dependent functional status			4.5 (1.5–13)	.006		
Consciousness/ cognitive impairment in background	52 (2.9–906)	.007	31.2 (3.6–274)	.002		
Malignant tumor (active or in background)	12.5 (5.3–30)	<.001	16.4 (7.8–34)	<.001		
Number of prior SUTI event (in previous year)					.7 (.6–.8)	<.001

Notes. ESBL, extended-spectrum beta-lactamase-producing Enterobacterales; CI, confidence intervals; MDRO, multidrug-resistant organism; SUTI, symptomatic urinary tract infection.

### Efficacy analyses of oral therapeutics used for CO-ESBL SUTI

There were 331 patients with CO-ESBL SUTI, who were managed with a single oral agent, for which the ESBL isolate was susceptible (in vitro). As depicted in Table 4, fosfomycin had the lowest resistance rates to ESBL strains (4.9%), while TMP/SMX (64%) and fluroquinolones (46%) had the highest resistance rates. The outcomes of patients with ESBL SUTI were generally unfavorable, and overall, 91 patients (28%) had met the composite worse outcome definition. The agent with the lowest composite outcome rate (22%) was TMP/SMX, while the agent with the worse composite outcome rate (32%) was amoxicillin-clavulanate.

**Table 4.** Resistance rates and effectiveness of oral antimicrobials used for treatment of community-onset (CO) extended-spectrum beta-lactamase-producing Enterobacterales (ESBL) symptomatic urinary tract infections (SUTI), Maccabi health care, 10–11/2019

	Pathogen resistance		Bacteriological failure^b^		Clinical failure^c^		ER referrals during the acute illness		Hospitalization in acute-care facility		Composite end point^d^	
	No.	Valid %^a^	No.	Valid %^a^	No.	Valid %^a^	No.	Valid %^a^	No.	Valid %^a^	No.	Valid %^a^
Case type	Among 485 ESBL isolates		Among 311 patients with CO-ESBL SUTI managed with single oral agents									
Fosfomycin (n = 102)	24	4.9	5	56	30	71	1	1	0		32	31
Nitrofurantoin (n = 83)	133	27	3	38	20	77	0		0		22	27
FQ (n = 103)	224	46	8	80	22	60	4	4	1	1	25	24
Amoxicillin-clavulanate (n = 25)	185	38	4	67	7	78	3	12	3	12	8	32
TMP-SMX (n = 18)	311	64	0		4	67	1	6	1	6	4	22

Notes. ER, emergency room; FQ, Fluoroquinolones (eg, ciprofloxacin, ofloxacin, levofloxacin); TMP-SMX, trimethoprim-sulfamethoxazole.

a Valid percent: count divided by the total number of valid observations (after excluding the missing information from the denominator).

b Bacteriological failures: continued positive urine culture within 14 days following culture date.

c Clinical failures: continued SUTI’s symptoms or complains documented within 14 days following culture date.

d Composite end point: bacteriological failure, clinical failure, referred to the emergency room, or had to be hospitalized.

## Discussion

ESBL SUTI is a common ambulatory infection, and the rapid dissemination of ESBL-producing strains in the community, as declared by the WHO,^5^ underscores the substantial burden of antimicrobial resistance on the general population. In this large study (1,455 patients), numerous potential predictors of CO-ESBL SUTI, were identified through bivariable (Table 1) and multivariable (Table 3) analyses. However, after applying the case-case-control design,^13^ most predictors were associated with CO SUTI in general, with recent hospitalization remaining the only independent predictor specifically linked to CO-ESBL SUTI. In the case-case analysis (ie, with SUTI patients reflecting the background population), recent hospitalization was an independent predictor for ESBL SUTI, along with MDRO carriage from the past two years, recent antimicrobials exposure, and the number of prior SUTI events from the past year (captured as a continuous variable). We recommend that clinicians in this Israeli region consider the possibility of ESBL infection when managing patients with mild CO SUTI who present with any of these four predictors.

As previously reported in hospital settings,^9,10^ community patients with CO-ESBL SUTI experienced significantly longer DAAT compared with those with CO-non-ESBL SUTI (ie, *P* < .001, Table 2). The increased DAAT, among other factors,^11^ might have resulted significant worse outcomes among patients with CO-ESBL SUTI (Table 2), but this was not one of the study aims and was not analyzed directly . Although none of the 1,455 patients died within 14 days of the index visit (ie, reflecting a population suffering a mild infectious syndrome), 150 of the CO-ESBL SUTI patients (31%) had met the composite definition of worse outcome, ie, implying SUTI treatment failure. This high rate of treatment failure underscores the epidemiologic importance of ESBL SUTI managed in community settings.

The comparative efficacy analysis between the oral regimens used to manage SUTI patients (Table 4), provides “real-world” descriptive data, from 331 patients with CO-ESBL SUTI,^1^ despite the relatively small sample size in each treatment arm. As emphasized in recent IDSA treatment guidelines, this field remains characterized by lack of controlled evidence.^17^ The outcomes of patients with ESBL SUTI did not differ between the various single oral agents, with nearly one-third of the patients experiencing treatment failure (Table 4). IDSA guidelines recommend to manage mild SUTI (regardless ESBL production) with either TMP/SMX or nitrofurantoin, as long as the offending isolate is susceptible.^17^ TMP/SMX indeed displayed the lowest treatment failure rates (22%, with none of the patients experiencing bacteriological failures), but it had the highest initial resistance rates (64%). Nitrofurantoin was associated with composite outcomes rate of 27%, in similar to the other agents (Table 4). The IDSA guidelines also recommend,^17^ and this is further supported by this study, that amoxicillin–clavulanate should be avoided for ESBL SUTI, even in mild infections caused by phenotypically susceptible strains.^23^ The outcomes of patients managed with amoxicillin-clavulanate were the worse among this cohort of patients (32%). Hiding from non-trained prescribers the susceptibility results to amoxicillin-clavulanate should be locally considered. The IDSA guidelines also discourages the usage of fluoroquinolones for mild SUTI,^17^ and indeed 46% of the ESBL offending isolates were a priori resistant (Table 4), in addition to a strong significant association between recent fluoroquinolone usage (3 months) and ESBL SUTI emergence (Table 1). Fosfomycin was commonly prescribed (102 patients), having significant lower resistance rate compared to other agents (4.9%; *P* < .001). Of note, fosfomycin usage in this study was in three consecutive doses (3 g), administered for three consecutive days.^18^ Despite the low resistance rates (resulting in part from the high benchmark to define non-susceptibility^23^) and the enhanced regimen of 9 g, fosfomycin treatment outcomes were unfavorable (31% meeting the composite outcome definition), as other oral agents. Of note, treatment failure rate among the 8 patients with a fosfomycin susceptible *K. pneumoniae* ESBL strain, treated with fosfomycin (33%), were similar to the overall treatment failures rates.^17^

Our study has several limitations. As an observational, retrospective, chart-review-based investigation, some documentation was missing or inaccurate. However, there is no reason to assume that these biases differentially affected either group, whether in the CO-ESBL SUTI predictors’ analysis (study aim 1) or in the oral agents’ effectiveness analysis (study aim 2). Since the study was executed in a single district from a single country, over a 2-month season, the results cannot be generalized to other locales without validation. However, the case-case-control and the case-case designs applied in this study (study aim 1), executed in community settings, applying established matching processes,^24^ and using two methods of analyses to explore independent predictors for CO-ESBL SUTI (to assist clinicians in the community in shortening DAAT), is a methodological strength. Additional limitation, in the treatment effectiveness sub analyses (study aim 2), are the low number of patients subjected to each treatment arm. We used a composite worse outcome, uniting several outcomes that represent SUTI treatment failures, to increase statistical power, acknowledging the natural complexities of capturing outcomes of a mild infectious syndrome, in chart-review retrospective study. Additional limitation, is the fact that outcomes were compared between groups without factoring severity of illness indices (who were commonly subtle ie Table 1), and other parameters that might affected patients’ outcomes. However, the data still project interesting descriptive (non-controlled) information, pertaining to the overall low cure rates of mild ESBL SUTI managed in the community, specifically patients managed with amoxicillin-clavulanate.

To conclude, CO-ESBL SUTI should be suspected, and empirically covered by primary prescribers in the Shfela district, Israel, particularly in patients with recent hospitalization, MDRO carriage, recent antimicrobials exposure, or prior SUTI events. This could reduce DAAT, which was frequent among patients with CO-ESBL SUTI, and improve their outcomes (which were significantly worse). In addition, according to this observational investigation, it is safe to manage CO-ESBL SUTI patients, who are not severely ill at the initial clinical assessment, with either TMP/SMX (although resistance rates are high) or any other agent, avoiding amoxicillin-clavulanate, even when the ESBL isolate is susceptible (in vitro). Further studies are needed to generalize these findings to other settings.
